# Characterization of bacteriophage communities and CRISPR profiles from dental plaque

**DOI:** 10.1186/1471-2180-14-175

**Published:** 2014-06-30

**Authors:** Mayuri Naidu, Refugio Robles-Sikisaka, Shira R Abeles, Tobias K Boehm, David T Pride

**Affiliations:** 1 Department of Pathology, University of California, San Diego, CA, USA; 2 Department of Medicine, University of California, San Diego, CA, USA; 3 College of Dental Medicine, Western University of Health Sciences, Pomona, CA, USA

**Keywords:** Oral biofilm, Virus, Virome, Microbiome, Dental plaque, CRISPR

## Abstract

**Background:**

Dental plaque is home to a diverse and complex community of bacteria, but has generally been believed to be inhabited by relatively few viruses. We sampled the saliva and dental plaque from 4 healthy human subjects to determine whether plaque was populated by viral communities, and whether there were differences in viral communities specific to subject or sample type.

**Results:**

We found that the plaque was inhabited by a community of bacteriophage whose membership was mostly subject-specific. There was a significant proportion of viral homologues shared between plaque and salivary viromes within each subject, suggesting that some oral viruses were present in both sites. We also characterized Clustered Regularly Interspaced Short Palindromic Repeats (CRISPRs) in oral streptococci, as their profiles provide clues to the viruses that oral bacteria may be able to counteract. While there were some CRISPR spacers specific to each sample type, many more were shared across sites and were highly subject specific. Many CRISPR spacers matched viruses present in plaque, suggesting that the evolution of CRISPR loci may have been specific to plaque-derived viruses.

**Conclusions:**

Our findings of subject specificity to both plaque-derived viruses and CRISPR profiles suggest that human viral ecology may be highly personalized.

## Background

Much of the study of the human microbiome has concentrated on those indigenous bacterial communities inhabiting different body surfaces [1-4], but relatively little effort has been focused on viruses [5-9]. Recent studies have identified communities of viruses inhabiting the human oral cavity [10,11], the respiratory tract [8], skin [12], and the intestinal tract [5,7,13]. While the role of viruses in these communities has yet to be thoroughly examined, a common feature shared among these body surfaces has been that most of the viruses identified have been bacteriophage [5-7,11,14]. Because bacteria generally outnumber human cells in these environments, bacteriophage might also be expected to outnumber eukaryotic viruses. Many of the viruses present in these communities have been predicted to have primarily lysogenic lifestyles, carrying gene function that might facilitate the pathogenic functions of their host bacteria [6,7].

Biofilms contain complex aggregates of microorganisms growing on self-produced solid surfaces, whose constituents and cellular activity may differ substantially from planktonic communities [15]. The oral biofilm is known to be inhabited by numerous species of bacteria and archaea [1,16-18], but has not been shown to be inhabited by communities of viruses. Because of the potential difficulty in traversing solid surface biofilms, dental plaque has been hypothesized to be relatively devoid of viruses [6], however, some viruses have previously been identified in dental plaque [19-21]. Given the abundance of bacteria residing within plaque, we hypothesize that dental plaque may have an indigenous viral community.

The human oral cavity contains many microenvironments in which the microbiota are known to differ [17]. There are characteristic differences in the relative abundances of bacteria in subgingival plaque, supragingival plaque, saliva, buccal mucosa and on the tongue. There also are shifts in oral bacteria that can be traced to diet [22] and oral health status [23-26]. Because of the proximity to tooth surfaces, many have sought to characterize subgingival microbiota in conditions such as chronic periodontal disease [27,28] and dental caries [29], as those communities harbor microbes that might contribute to oral inflammation and the subsequent development of disease. Whether viral communities are part of the biofilm microbiota or contribute to oral inflammation has not previously reported.

Characterization of human viral communities has generally been limited by a relative dearth of homologous sequences available to identify metagenome contents [10,30,31]. Most of the studies characterizing human viral communities have viromes in which greater than half of the constituents are without homologues [5,6]. Other studies have used Clustered Regularly Interspaced Short Palindromic Repeats (CRISPRs) in bacteria, which acquire short sequences from the viruses to which they are exposed [32-34], as a means to augment analysis of human viral communities. Some dental plaque biota are known to possess CRISPR/Cas systems [35], suggesting that they can adapt to invading viruses. We believe that there are uncharacterized populations of viruses inhabiting the oral biofilm that may have unique features when compared to planktonic viruses in saliva. In this study, we sought to detail the presence of viral communities populating dental plaque, to determine whether oral viruses might be subject specific or specific to oral sampling site, and to characterize the potential capacity of oral streptococci to counteract their viruses by profiling CRISPRs.

## Results

### Isolation and sequencing of dental plaque viromes

Although some viruses have previously been isolated [19-21], it is not known whether dental plaque is inhabited by a community of viruses as has been shown for saliva [6,10,11]. To determine whether there existed a population of viruses in dental plaque, we evaluated plaque from 4 human subjects with good overall periodontal health (Additional file 1: Table S1). We collected plaque in a biogeographic manner from tooth #3, 9, 12, 19, 25, and 28 (see Additional file 1: Table S2 for international numeration). Virus-like particles (VLPs) were visualized from dental plaque using epifluorescence microscopy and were present at an estimated 10^10^ VLPs per gram of plaque for all subjects (Additional file 2: Figure S1). Comparatively, there were 10^8^ VLPs per ml of saliva in these same subjects, 10^8^ in the lower respiratory tract of other human subjects, 10^5^ in blood, 10^7^ in the vagina, and 10^8^ in the human gut virome [36].

Viromes were enriched from the dental plaque of each subject similar to our previously described protocols for isolating DNA viruses from saliva [10]. We sequenced 7,768,251 virome reads from all subjects (3,181,703 from saliva and 4,586,548 from dental plaque) using semiconductor sequencing [37]. All viromes were screened for contaminating cellular nucleic acids by BLASTN analysis against a human reference database and a composite database of 16S rRNA. No homologues were identified among the viromes to 16S rRNA, indicating that these viromes were relatively free of contaminating bacterial DNA (Additional file 1: Table S3). A small number of reads homologous to human DNA were identified in the dental plaque virome of subject #3 (721 reads represented 0.06% of the virome reads), and were removed prior to further analysis.

### Characterization of plaque viromes

**Figure 1:**
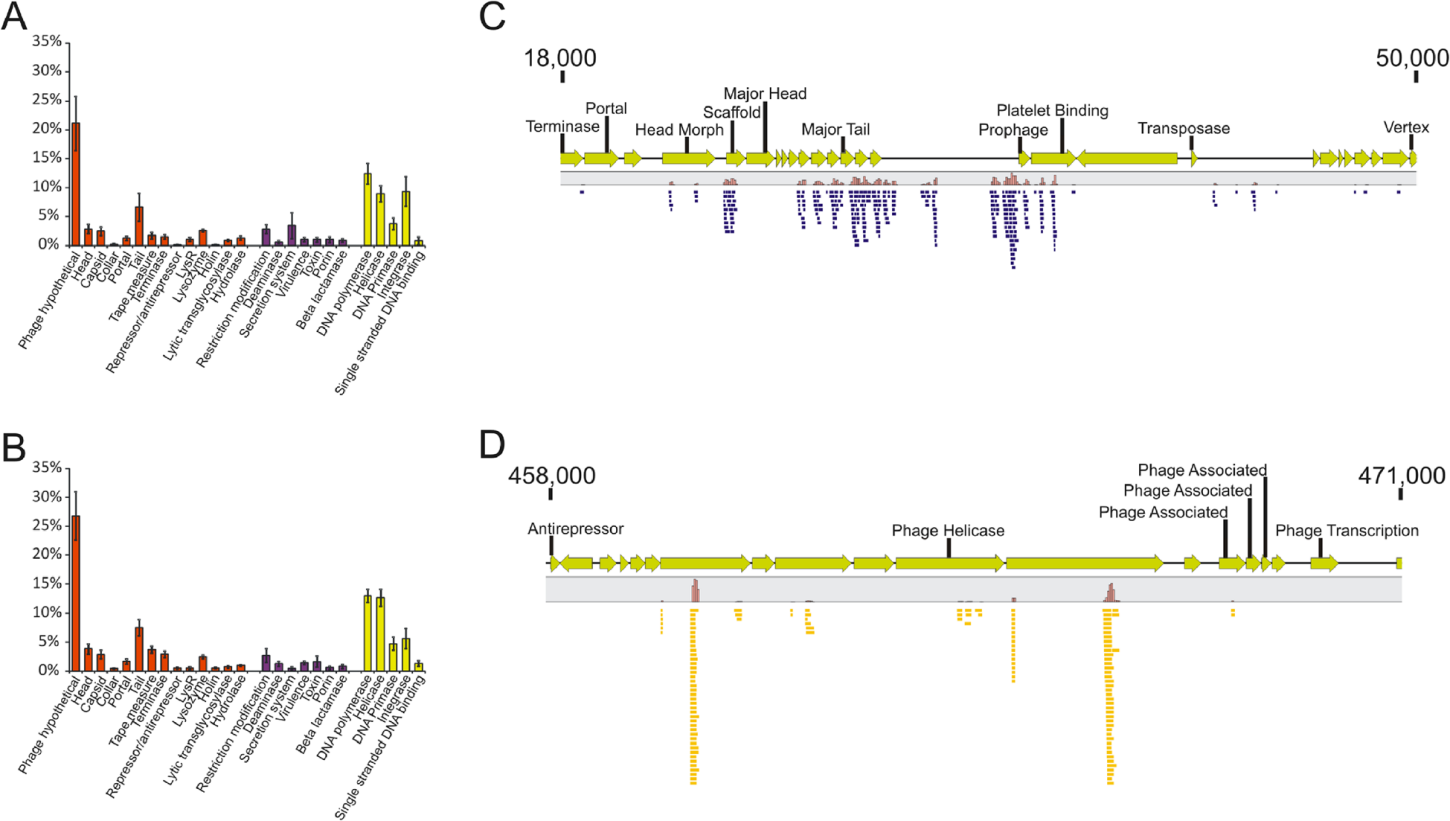
**Percentages of contigs with viral homologues (Panels A and B) and mappings of virome reads to select bacterial genomes (Panels C and D).** Homologues to genes involved in virulence are represented in purple, replication and integration in yellow, and all others including structural and hypothetical genes in orange. Contigs from saliva are shown in Panel **A** and contigs from dental plaque are shown in Panel **B**. Read mappings of virome reads from subject #4 to *Streptococcus pseudopneumoniae IS7493* is shown in Panel **C** and read mappings from subject #3 to *Streptococcus gallolyticus UCN34* is shown in Panel **D**. Putative ORFs are represented by yellow arrows and the annotation provided above each ORF. Those ORFs without annotation represent hypothetical coding sequences. The relative proportion of reads and location where the reads map is demonstrated in blue in Panel **C** and gold in Panel **D**. Coordinates within each genome also are demonstrated at the top of each diagram.

**Figure 2:**
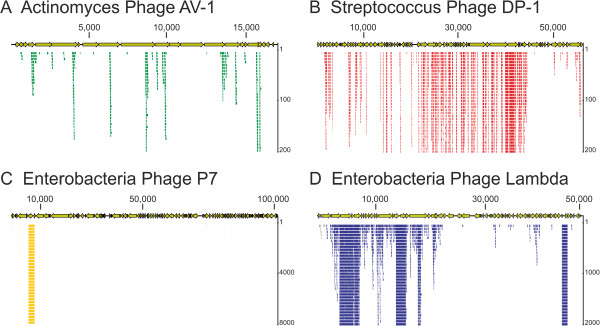
**Mappings of virome reads from each subject to select viruses.** Panel **A** - the virome read mappings from subject #1 dental plaque to Actinomyces phage AV-1, Panel **B** - the virome read mappings from subject #2 to Streptococcus phage DP-1, Panel **C** – the virome read mappings from subject #3 to Enterobacteria phage P7, and Panel **D** – the virome read mappings from subject #4 to Enterobacteria phage Lambda. The *y*-axis demonstrates the total number of reads mapping to individual segments of each virus.

To characterize the viral populations present in dental plaque, we assembled the virome reads from each subject and sample type, and searched the NCBI NR database for homologous sequences. A substantial proportion of each virome was homologous to known viruses (Additional file 2: Figure S2), with >99% of the viral contigs representing bacteriophage. Circoviruses and herpesviruses were the only human viruses identified, and each represented only a minority of the population. The distribution of structural, virulence, and replication genes amongst the bacteriophage present was similar for both saliva and dental plaque, where the most commonly identified phage genes were polymerases, helicases, integrases, tail fibers, and hypothetical genes in both sample types (Figure 1, Panels A and B). Many virome contigs had no known homologues, while others were homologous to bacterial genomes. Further analysis of these viromes demonstrated that many of the sequences identified as homologous to bacteria were actually homologous to un-annotated phage or hypothetical genes within prophage in bacterial genomes. For example, many of the reads from subject #3 map to a small segment of *Streptococcus gallolyticus UCN34* (Figure 1, Panel C), which represents a prophage. Similar findings were found for subject #4, where many of the reads map to un-annotated genes in a prophage within the *S. pseudopneumoniae IS7493* genome (Figure 1, Panel D). As many of the genes in these prophage were not annotated, they appeared as homologues only to the bacterial genomes. There were few reads in either virome that mapped to portions of *S. gallolyticus* or *S. pseudopneumoniae* genomes outside of these prophage. Reads from each subjects and sample type also mapped specifically to the CRISPR loci of *S. gordonii* challis CH1 (Additional file 2: Figure S3) and 3 separate *S. thermophilus* isolates (Additional file 2: Figure S4). None of these virome reads had any identifiable CRISPR repeat motifs, which further supports that they were viral in origin rather than from bacteria. All of the CRISPR spacers in *S. gordonii* challis CH1 matched virome reads from subject #1 and #4, indicating that viruses matched by those CRISPR spacers were prevalent in those subjects.We also compared the viromes from each subject to a database of known bacteriophage to determine whether similar phage might have been present in the oral cavities of each subject. Many reads mapped to Actinomyces phage AV-1 from dental plaque in subject #1 (Figure 2, Panel A), to Streptococcus phage DP-1 in subject #2 (Panel B), to Enterobacteria Phage P7 in subject #3 (Panel C), and to Enterobacteria Phage Lambda in Subject #4 (Panel D). Over 6% (71,945 of 1,164,502 reads) of the virome from the plaque in subject #3 mapped to a short segment of Enterobacteria Phage P7 containing a transposon encoding tetracycline resistance.

### Viral and bacterial community composition by subject and sample type

**Figure 3:**
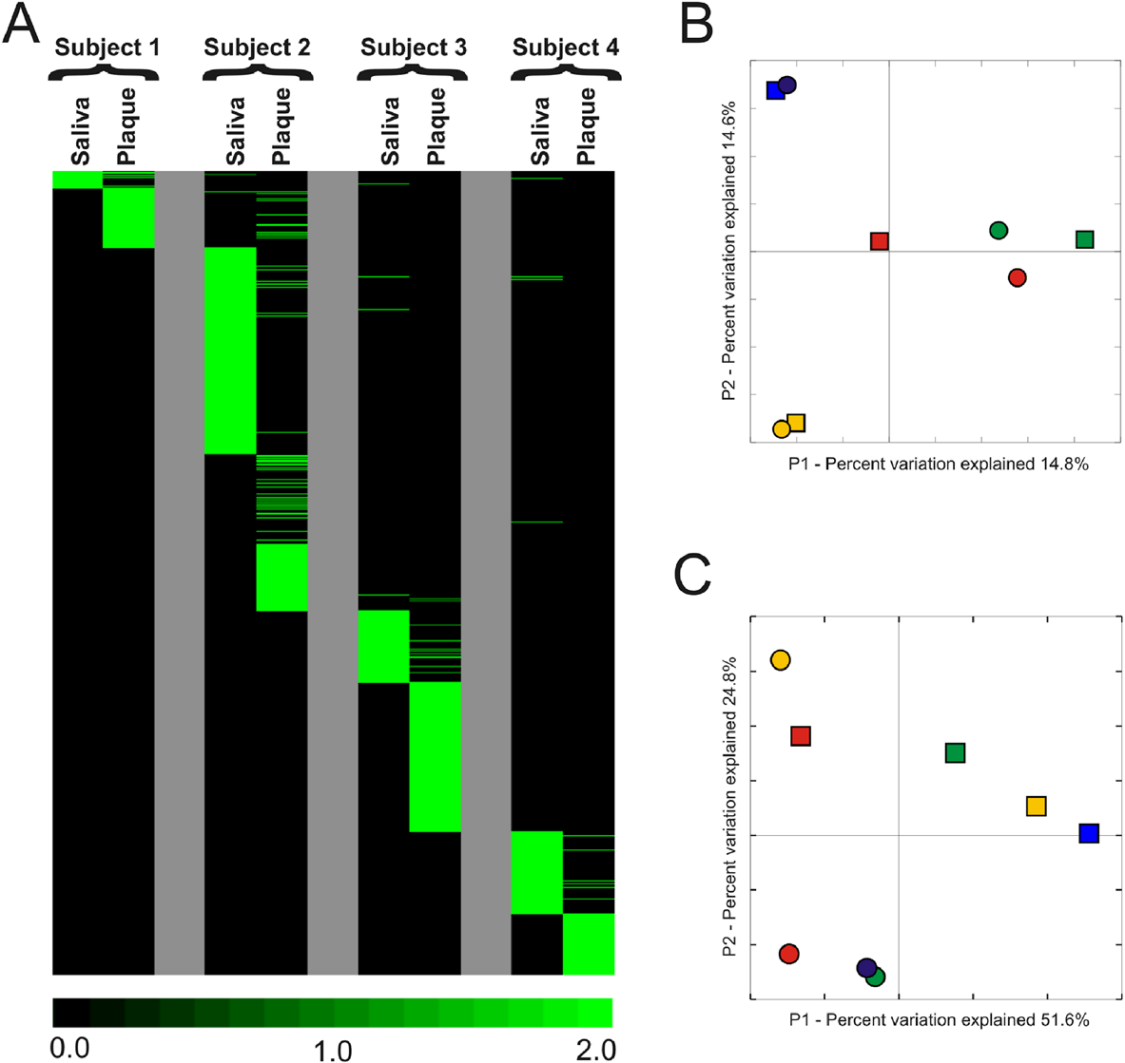
**Heatmap of virome contigs (Panel A) and principal coordinates analysis of virome contigs (Panel B) and bacteria 16S rRNA (Panel C) from each subject and biogeographic site.** Panel **A** - Each row represents a unique homologue, and the columns represent viromes from each subject and sample type. The intensity scale bar is located below the heatmap. In Panels **B** and **C**, subject #1 is represented in green, subject #2 in red, subject #3 in gold, and subject #4 in blue. Saliva is represented by squares and dental plaque by circles.

We compared the constituents of each virome to determine whether there were characteristics specific to each subject or sample type. We found some viral contigs that were homologous across all subjects, indicating that viruses sharing similar sequence features were present in each subject and sample type (Figure 3, Panel A). We used principal coordinates analysis to determine whether virome composition might be influenced by subject or sample type. Both the dental plaque and saliva viromes were highly reflective of their host environment (Figure 3, Panel B).

We also characterized the bacterial community composition in each subject and sample type by analysis of the V3 region of 16S rRNA. We sequenced 190,720 reads (average of 15,893 per subject and site) from each subject and sample type (Additional file 1: Table S4). Rarefaction analysis demonstrated that the preponderance of bacterial diversity had been sampled in each subject and sample type (Additional file 2: Figure S5). Contrary to the subject-specific results found for viruses in the oral cavity (Figure 3, Panel B), sample type was an important determinant of oral bacterial ecology (Figure 3, Panel C).

We quantified the proportion of homologous reads between viromes to determine whether patterns of variations observed in principal coordinates analysis were statistically supported. Using a permutation test, there was substantial intra-subject homology between saliva and dental plaque (range 45-74%). The proportion of intra-subject shared viral homologues were statistically significant for subjects #1, #2, and #4 (Table 1). There also was significant homology for inter-subject comparisons of dental plaque (p = 0.05), but was not observed for saliva. These data indicate that both sample type and individual host environment were important determinants of oral viral ecology.

**Table 1:** Viral homologues between subjects and sites

	**Percent homologous within subject or sample type**^**a**^	**Percent homologous for comparisons of different subjects or sample types**^**a**^	**P value**^**b**^
**By subject**			
Subject 1	74.11 ± 5.55	36.52 ± 19.80	**0.0122**
Subject 2	67.14 ± 3.80	37.76 ± 16.87	**0.0432**
Subject 3	44.59 ± 6.75	40.26 ± 19.19	0.4461
Subject 4	54.26 ± 6.20	36.37 ± 19.92	**0.0511**
**By sample type**			
Saliva	57.50 ± 8.01	56.94 ± 13.92	0.5339
Plaque	72.94 ± 3.23	61.00 ± 10.00	**0.0522**

^a^Based on the mean of 10,000 iterations. 10,000 random reads were sampled per iteration.

^b^Empirical p-value based on the fraction of times the estimated percentage of homologous reads for each subject or sample type exceeds that for different subjects or sample types. P-values ≤0.05 or less are highlighted in bold.

### Streptococcal CRISPR profiles in dental plaque

We previously profiled streptococcal CRISPRs in the saliva of a cohort of human subjects and identified many matching viral sequences in those same subjects [6]. We evaluated the same Streptococcus Group I (SGI) and Streptococcus Group II (SGII) CRISPRs, both of which represent Type II CRISPR/Cas systems in each species [38]. These repeat motifs have been identified in numerous different streptococcal species (Additional file 1: Table S5) [6,35]. We sequenced 293,139 SGI and 229,103 SGII CRISPR spacers from each subject and sample type (Additional file 1: Tables S6 and S7), and binned spacers according to their trinucleotide content to account for any potential polymorphisms or sequencing errors [11]. When examining spacer content, only 0.002% of SGI and 0.001% of SGII CRISPR spacers were estimated to have any polymorphisms (Additional file 2: Figure S6).

**Figure 4:**
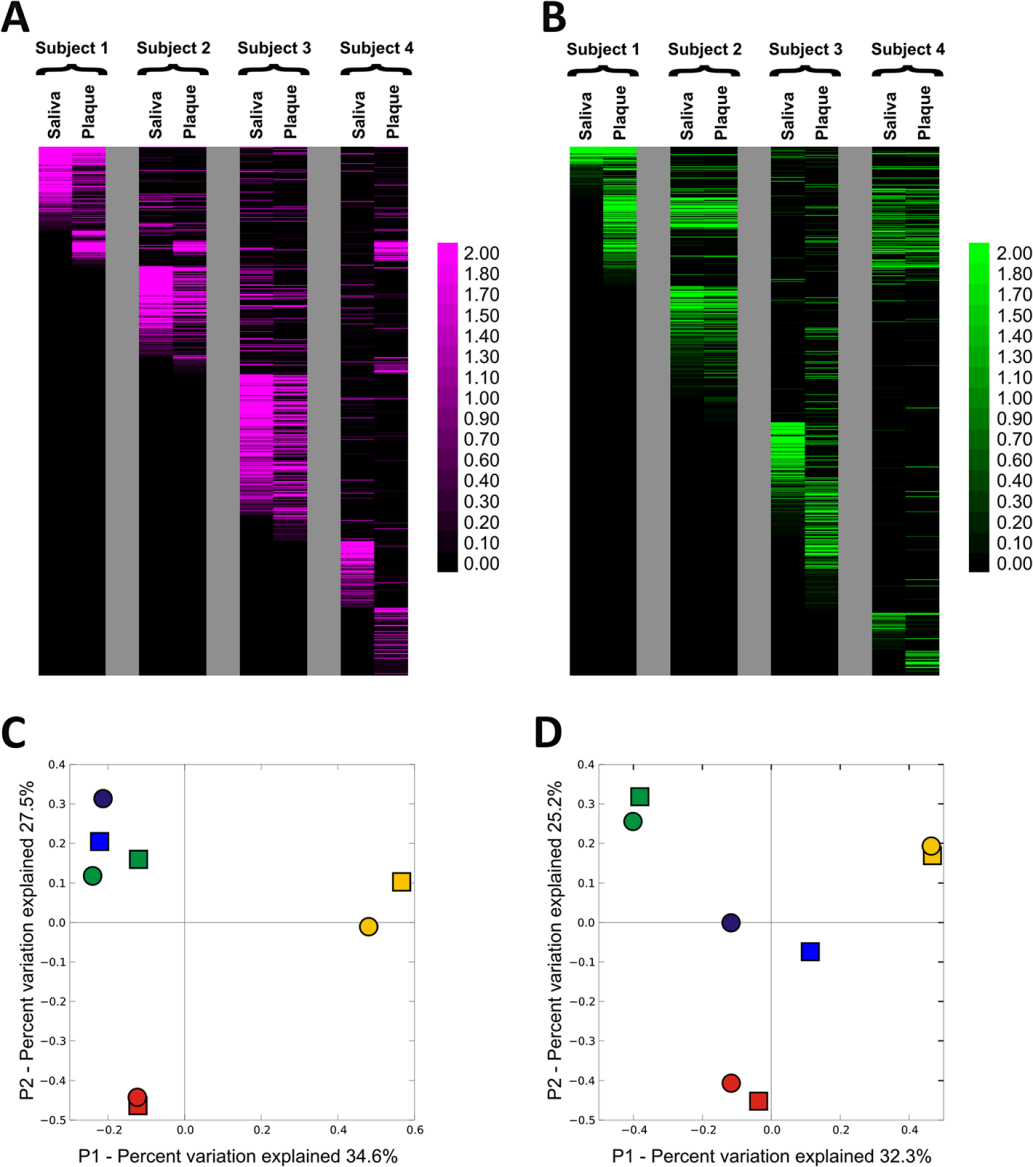
**Heatmap and principal coordinates analysis of SGI (Panels A and C) and SGII (Panels B and D) CRISPR spacer groups from all subjects and sample types.** Panels **A** and **B** - Each row represents a unique CRISPR spacer group, and the columns represent each subject and biogeographic site. The intensity scale bar is located to the right of each heatmap. Panels **C** and **D** - Principal coordinates analysis of CRISPR spacer groups. Subject #1 is represented in green, subject #2 in red, subject #3 in gold, and subject #4 in blue. Saliva is represented by squares and plaque by circles.

We examined the distribution of CRISPR spacers to determine whether similar spacer profiles were present in each subject and sample type. For each subject, there were SGI and SGII spacers shared between plaque and saliva, but there also were some that were unique to each sample type (Figure 4, Panels A and B). The patterns of variation observed in CRISPR spacers were highly reflective of their host environment for both SGI and SGII spacers (Figure 4, Panels C and D), similar to results found for viromes (Figure 3, Panel B; Table 1). We also quantified the level of shared spacers between subjects. When the relative abundance of spacer sequences was considered, there was a significant (p < 0.05) proportion of shared spacers within each subject (71% to 97% for SGI and 89% to 99% for SGII), with the exception of subject #4 SGI CRISPRs (Table 2). No significant proportions of shared CRISPR spacers were found when compared by oral sample type.

**Table 2:** CRISPR spacer groups shared between subjects and sites

	**Percent shared within subject or sample type**^**a**^	**Percent shared for comparisons of different subjects or sample types**^**a**^	**P value**^**b**^
**SGI CRISPRs**			
**By subject**			
Subject 1	96.74 ± 0.63	50.20 ± 22.18	**0.0011**
Subject 2	94.95 ± 0.73	50.00 ± 22.22	**0.0122**
Subject 3	96.51 ± 0.50	50.07 ± 22.24	**0.0015**
Subject 4	71.68 ± 4.22	50.23 ± 22.28	0.2256
**By sample type**			
Saliva	14.12 ± 3.64	23.93 ± 5.27	0.9541
Plaque	21.35 ± 3.89	15.77 ± 4.58	0.4491
**SGII CRISPRs**			
**By subject**			
Subject 1	95.79 ± 0.70	60.90 ± 19.27	**0.0064**
Subject 2	99.48 ± 0.06	60.94 ± 19.20	**0.0014**
Subject 3	95.80 ± 0.56	60.67 ± 19.26	**0.0065**
Subject 4	89.16 ± 1.76	60.85 ± 19.23	**0.0495**
**By sample type**			
Saliva	15.84 ± 3.40	32.88 ± 4.26	0.9998
Plaque	25.64 ± 2.88	20.66 ± 3.44	0.4869

^a^Based on the mean of 10,000 iterations. 1,000 random spacers were sampled per iteration.

^b^Empirical p-value based on the fraction of times the estimated percentage of shared spacer groups for each subject or sample type exceeds that for different subjects or sample types. P-values ≤0.05 are highlighted in bold.

### CRISPR spacers from dental plaque match oral viruses

**Figure 5:**
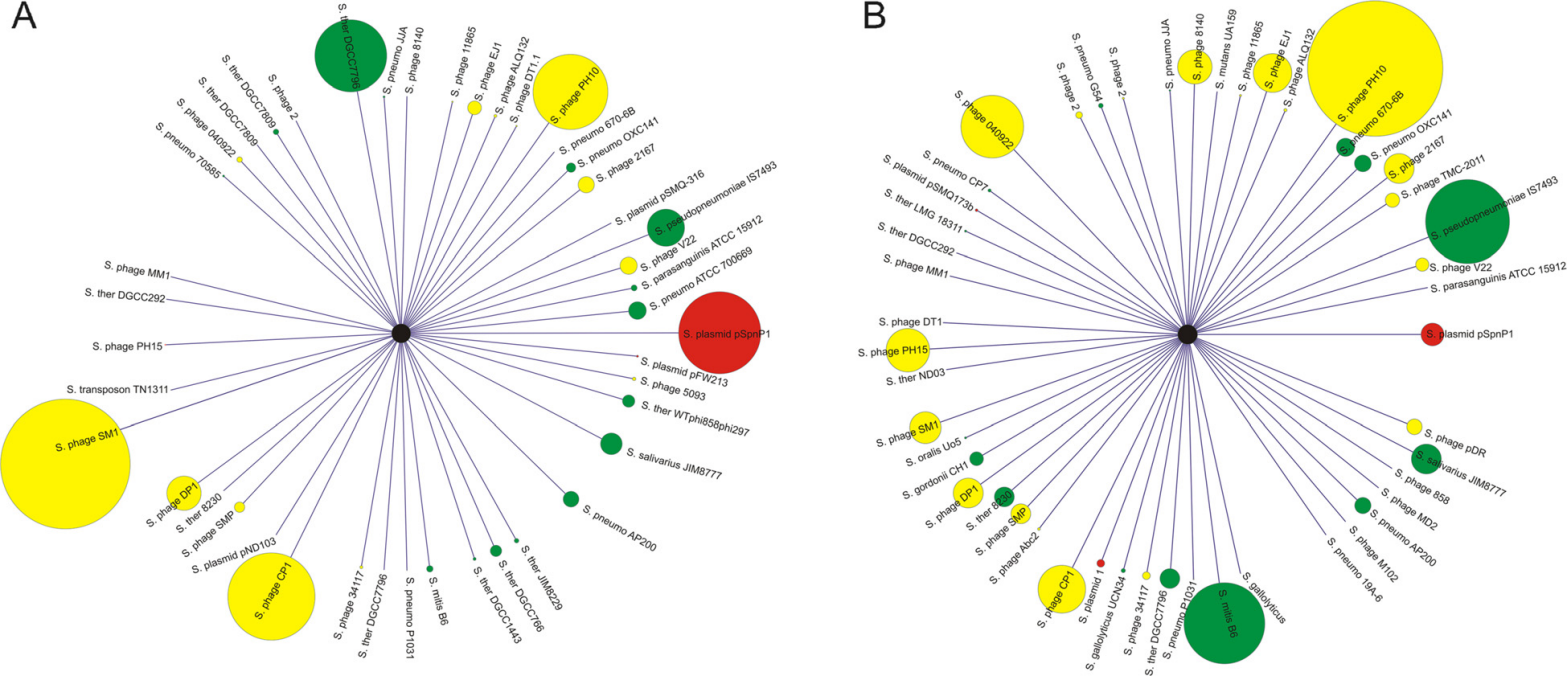
**Radial diagram of SGI (Panel A) and SGII (Panel B) CRISPR spacer groups with streptococcal homologues.** The relative number of CRISPR spacer groups homologous to each sequence is drawn to scale. Yellow represents streptococcal viruses, green represents streptococcal genomes, and red represents streptococcal plasmids.

**Figure 6:**
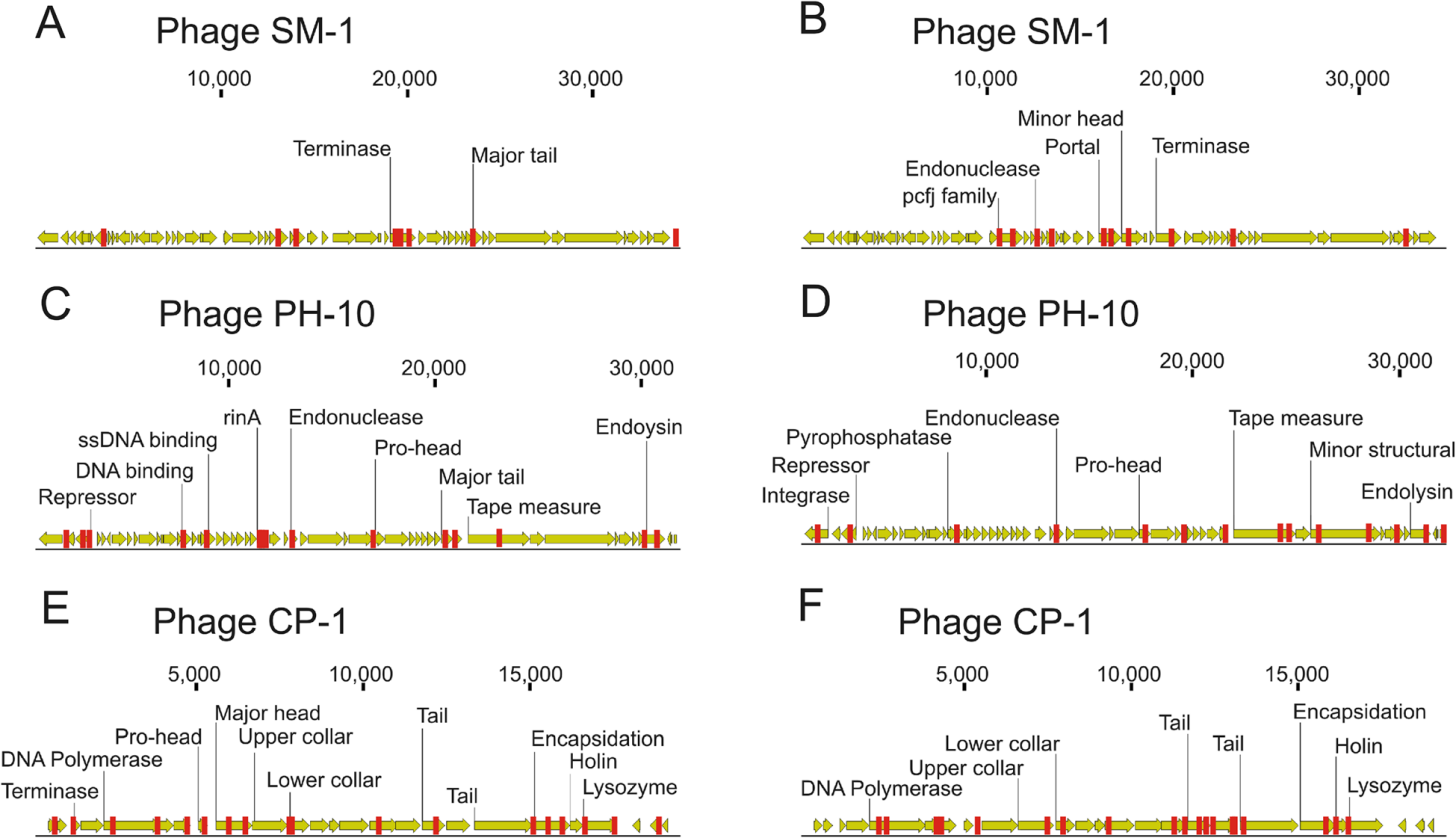
**Diagram of CRISPR spacers with exact matches and their locations along the genomes of several streptococcal bacteriophage.** SGII CRISPR spacer mappings are shown in Panels **A**, **C**, and **E**, while SGI CRISPR spacer mappings are shown in Panels **B**, **D**, and **F**. Bacteriophage SM-1 is shown in Panels **A** and **B**, phage PH-10 is shown in Panels **C** and **D**, and phage CP-1 is shown in Panels **E** and **F**. The genes in each phage and their orientation are shown in yellow, and matches to each gene and their relative locations along each gene are shown in red. Putative functions assigned to each gene are demonstrated above each gene, and the relative length of each phage is shown at the top of each panel.

**Figure 7:**
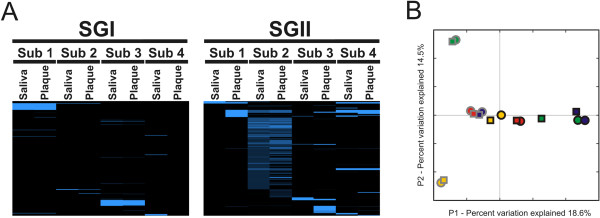
**Heatmap (Panel A) and principal coordinates analysis (Panel B) of CRISPR spacer-virome read matches for all subjects and sample types.** Each heatmap row represents reads from the viromes from each subject, and columns represent each subject and sample type. In Panel **B**, subject #1 is represented in green, subject #2 in red, subject #3 in gold, and subject #4 in blue. Saliva is represented by squares and plaque by circles. Grey outlines represent SGI CRISPR spacers and black outlines represent SGII CRISPR spacers.

We tested whether the SGI and SGII CRISPR spacer sequences had homologues in the NCBI NR database, and found many homologous to streptococcal viruses, genomes, and plasmids in each subject and sample type (Additional file 1: Table S8). While none of the SGI and SGII spacers were identical, many had exact matches to the same streptococcal viruses and plasmids (Figure 5). Streptococcus phage SM-1 (Figure 6, Panels A and B), PH-10 (Panels C and D), and CP-1 (Panels E and F) were amongst the most highly matched viruses by CRISPR spacers from dental plaque. Different portions of the same genes in these phage were matched by both SGII and SGI spacers. For example, in phage PH-10, the repressor, endonuclease, pro-head, tape measure, and endolysin were all matched by SGII (Panel C) and SGI (Panel D) spacers derived from plaque. We also mapped SGI and SGII CRISPR spacers to the genomes of many oral streptococci and also found exact matches to putative prophage in streptococcal genomes. For example, both SGI and SGII CRISPRs matched a known prophage in *S. mitis* B6 and multiple prophage in *S. pneumoniae* 670-6B (Additional file 2: Figure S7). Many of these matches were derived from plaque-derived CRISPR spacers (Additional file 1: Table S8) and occurred across the genome sequences of each prophage.To determine whether CRISPRs from each sample type matched viruses from each subject, we compared virome and CRISPR data. Matches to virome reads were defined as exact matches to any spacer within a spacer group. Because the percentage of virome read/spacer matches was low, we combined viromes from all subjects prior to the analysis. We found that there were numerous SGI and SGII spacers that matched virome reads from the oral biofilm (Figure 7, Panel A). We also examined the patterns of CRISPR spacer/virome read matches to determine whether there was evidence for subject- or sample type-specific patterns. The patterns of spacer/virome matches observed reflected subject but not sample type specificity (Figure 7, Panel B). The CRISPR spacer data were complimentary to the observed subject-specific patterns observed in viromes.

## Discussion

Our analysis of the viral communities in dental plaque provides insights into relatively unexplored aspects of the microbiota inhabiting the complex oral ecosystem. While the relative paucity of biomass at each tooth precluded analysis of individual teeth, the pooling of dental plaque allowed for analysis of the viruses present. The sampling and analysis of the microbiota in dental plaque and saliva has been performed and reported on for many years [39-41], and the overlap in the viral communities observed between each likely reflects some overlap in the resident bacterial biota from both sites. In support of this hypothesis is the substantial proportion of shared CRISPRs spacers that likely reflect sampling of the same bacteria from both sites in each subject (Figure 4).

The vast majority of the viruses found in this study and others describing human viromes [5,8-11] have been identified as bacteriophage, with only a few eukaryote viruses including herpesviruses and circoviruses identified. Characterization of bacteriophage from viromes generally has been limited due to a lack of available homologous sequences. The proportion of contigs without homologous sequences in this study was greater than 50% in some viromes, similar to proportions found in other studies [5,8-10]. We identified numerous homologues to known viruses (Additional file 2: Figure S2) and found that many spanned the entire genome sequences of known viruses (Figure 2), which reinforced that there likely were full-length viral genomes present in dental plaque. Further study with a broader group of participants would be required to define what role viruses may play as members of the dental plaque microbiome.

We explored both bacterial and viral ecology to provide a more comprehensive view of the microbial inhabitants of plaque. While viral ecology was reflective of the subject from which they were derived (Figure 3, Panel B), the bacterial ecology was more reflective of sample type (Panel C). The membership of the dental plaque viral communities differed from planktonic saliva in all subjects, although there were homologous sequences between saliva and plaque in each subject (Figure 3, Panel A; Table 1). The significant proportion of homologous sequences for intra-subject comparisons of viromes and for inter-subject comparisons of dental plaque, suggests that oral viral ecology is influenced by both individual host environment and sample type. There were a significant number of VLPs present in both saliva and dental plaque, which were greater than most other body surfaces. The substantial population of phage present in plaque combined with the high numbers colonizing mucosal surfaces [42], increases the complexity of comparing relative abundances of oral phage with their putative bacterial hosts.

We studied CRISPRs in the dental plaque of our cohort, as their spacer sequences reveal sequence features of viruses that oral bacteria may counteract. The similar CRISPR profiles in both saliva and plaque likely reflect shared bacterial inhabitants in both niches. The overall trend in shared CRISPR spacers reflected a subject-specific rather than a sample type specific pattern in all subjects (Figure 4, Panels C and D). The CRISPR and virome data together demonstrate distinct ecological differences between subjects, and supports that both oral biogeography and the individual host environment are significant determinants of oral viral ecology. We previously have identified short proto-spacer-adjacent motifs (PAMs) that are used to recognize and select spacers from invading DNA for both SGI and SGII spacers [6].

## Conclusions

As we continue to characterize human microbial communities, we must account for the complexities of biogeography and its potential contribution to an individual’s microbial ecology. Our analysis of dental plaque has uncovered the presence of a community of viruses, whose constituents share some overlap with those of planktonic saliva. Despite that many of the viral contigs identified were unique to either saliva or dental plaque, the overlap observed in the saliva and plaque of individual subjects suggest that there may be shared viruses across each biogeographic site. The analysis presented here provides an additional framework for understanding human oral viral ecology, and demonstrate that oral viruses may be relatively personal features of the human microbiome.

## Methods

### Subject enrollment and sample collection

Subjects were recruited and enrolled from the Western University College of Dental Medicine and were approved by the University of California, San Diego and the Western University Administrative Panels on Human Subjects in Medical Research. All subjects signed an informed consent demonstrating their willingness to participate in the study. Each subject underwent a baseline periodontal examination including measurements of probing depths, clinical attachment loss, Gingival Index, Plaque Index, and gingival irritation [43], and were all found to be periodontally healthy with no carious lesions. We used the 1999 International Workshop for Classification of Periodontal Diseases and Conditions, where periodontitis including juvenile forms of periodontitis is defined by loss of attachment. For diagnosis of healthy, all sites had to have an attachment level of 0 mm and an absence of bleeding on probing. We excluded attachment levels from sites that were located next to 3rd molars, edentulous areas and sites where attachment loss was clearly caused by factors other than periodontal disease such as chronic toothbrush trauma. Exclusion criteria included antibiotic administration during or for 12 months prior to the beginning of the study and preexisting medical conditions that could result in immunosuppression. Plaque samples were collected first, followed by the patient allowing saliva to pool in his or her mouth for about 5 minutes, followed by collection of pooled saliva into a test tube. Plaque collection was modeled after standard plaque collection procedures used to perform clinical microbial sampling. Teeth were isolated with a rolled sheet of gauze on either side of the tooth, and gently dabbed dry with another piece of gauze. Supragingival plaque was collected with a Gracey curette by scraping the cutting edge of the instrument against the mesial surface of the tooth from the gingival margin and coronal to that, collecting a strip of plaque from the mesiobuccal line angle toward the interproximal contact. For subgingival plaque sampling, the other end of the curette was used to collect plaque below the gingival margin from the mesiolingual line angle towards the contact point. We attempted to performed this process in less than ten seconds to limit exposure of the sample to ambient air. Plaque was collected from the subgingival and supragingival biofilms from tooth #3, 9, 12, 19, 25, and 28 and placed into 200 μl of 0.02-micron filtered phosphate-buffered saline (PBS) (Fisher Scientific, Chico, CA) (See Additional file 1: Table S2 for international enumeration of teeth). Approximately 3 ml of saliva was collected without stimulation from each subject. Both saliva and dental plaque specimens were immediately frozen on dry ice and stored at −80°C until use in this study.

### Isolation and analysis of oral viruses

Dental plaque was pooled together by subject, washed twice in 0.02-micron filtered PBS, and spun at 6,000 g for 10 minutes to pellet the biofilm. The biofilm then was incubated at 37°C for 30 minutes, and vortexed vigorously for 10 minutes to separate out viruses. The biofilm was then spun at 6,000 g for 10 minutes, and the supernatant kept for further analysis. A small portion (0.05 g) of the VLPs from each subject were resuspended in 200 μl of 0.02-micron filtered PBS and their counts per gram of plaque determined by epifluorescence microscopy [44]. The remaining supernatant samples then were treated in an identical manner to those of the saliva samples, according to previously described methods for enrichment and extraction of nucleic acids from viruses [10]. The resulting DNA was amplified using the GenomiPhi V2 MDA amplification kit (GE Healthcare, Pittsburgh, PA), fragmented to roughly 100 to 200 bp using a Bioruptor (Diagenode, Denville, NJ), libraries created using the Ion Plus Fragment Library Kit (Life Technologies, Grand Island, NY) according to manufacturer’s instructions, and sequenced using 314 chips on an Ion Torrent Personal Genome Machine (PGM; Life Technologies, Grand Island, NY) [37]. Each resulting read was trimmed according to modified Phred quality scores using CLC Genomics Workbench 4.65 (CLC bio USA, Cambridge, MA), and low complexity reads (where >20% of the length were due to homopolymer tracts), reads with substantial length variation (<50 nucleotides or >200 nucleotides), and reads containing ambiguous characters were removed prior to further analysis. Reads were screened for homology to a composite database of 16S rRNA including the Ribosomal Database Project database [45], Green Genes database [46] and Silva database [47] using BLASTN analysis with an E-score cutoff value of 10^−5^. Reads also were screened for homology to the Human Reference Database at (ftp://ftp.ncbi.nlm.nih.gov/genbank/genomes/Eukaryotes/vertebrates_mammals/Homo_sapiens/) by BLASTN analysis using an E-score cutoff value of 10^−5^. Any reads homologous to sequences in the human database were removed prior to further analysis. Reads then were assembled using CLC Genomics Workbench 4.65 (CLC bio USA, Cambridge, MA) to construct contigs based on 98% identity with a minimum of 50% read overlap, consistent with criteria developed to discriminate between highly related viruses [48]. Because the shortest reads were 50 nucleotides, the minimum tolerable overlap was 25 nucleotides, and the average overlap was no less than 50 nucleotides depending on the characteristics of each virome. Contigs <200 bp were removed from further study. Specific viral homologues were determined by parsing BLASTX results (E-score cutoff value of 10^−5^) for known viral genes including replication, structural, transposition, restriction/modification, hypothetical, and other genes previously found in viruses for which the E-score was at least 10^−5^. Heatmaps were created using JAVA Treeview [49] based on a database of BLASTX best hits for all virome contigs, and were normalized based on the total number of viral contigs for each virome. Analysis of shared homologues present in each virome was performed by creating custom BLAST databases for each virome, comparing each database with all other viromes using BLASTN analysis (E-score <10^−5^), and normalization to the size of the smaller virome. Principal coordinates analysis was performed on homologous virome reads with binary Sorensen distances using Qiime [50]. Read mapping of viromes to a combined database of viruses (http://www.phantome.org; ftp://ftp.ncbi.nih.gov/genomes/Viruses/) or to bacterial genomes was performed using CLC Genomics Workbench 4.65 (CLC bio USA, Cambridge, MA), and were mapped using 98% identity over a minimum of 50% of the read length. Many of the virome sequences mapped to CRISPR loci within bacterial genomes, but none matched the CRISPR repeat motifs.

### Amplification and sequencing of CRISPRs

From each subject, genomic DNA was prepared from saliva or pooled subgingival or supragingival plaque using the QIAamp DNA MINI Kit (Qiagen, Valencia, CA), with the addition of a bead beating step using Lysing Matrix B (MPBio, Solon, OH) prior to nucleic acid extraction. SGII Primers were designed based on their specificity to the CRISPR repeat motifs present in *S. gordonii* str. Challis substr. CH1, *S. thermophilus* LMD-9, *S. thermophilus* LMG-18311, and *S. thermophilus* CNRZ-1066, and SGI primers were designed based on their specificity to the CRISPR repeat motifs present in *S. mutans* UA159, *S. thermophilus* LMD-9, and *S. thermophilus* LMG-18311 (Additional file 1: Table S9). Each forward primer contained 10-nucleotide barcode sequences, represented by the ‘X’ in each primer sequence (Additional file 1: Table S10). Reaction conditions included 44 μl Platinum High-Fidelity PCR Mastermix (Invitrogen, Carlsbad, CA), 1 μl of each the forward and reverse primer (10 mmol each), and 4 μl DNA template. The following were used as cycling parameters: 2 minutes initial denaturation at 94°C, followed by 30 cycles of denaturation (15 seconds at 95°C), annealing (15 seconds), and extension (2 minutes at 72°C), followed by a final extension (10 minutes at 72°C). CRISPR amplicons were purified using the MinElute PCR Purification Kit (Qiagen, Valencia, CA) followed by magnetic bead purification using Ampure XP (Agencourt, Beverly, MA). Molar equivalents were determined from each product using a Bioanalyzer HS DNA Kit (Agilent, Santa Clara, CA), and each were pooled into equimolar proportions. Resulting pools were sequenced using an Ion Torrent PGM according to manufacturer’s instructions (Life Technologies, Grand Island, NY) [37]. Barcoded sequences were then binned according to 100% matching barcodes. Each read was trimmed according to modified Phred scores of 0.5 using CLC Genomics Workbench 4.65 (CLC bio USA, Cambridge, MA), and low complexity reads and reads with ambiguous characters were removed from the analysis. Only those reads that had 100% matching sequences to both the 5’ and the 3’ end of the CRISPR repeat motifs were used for further evaluation. Spacers were defined as any nucleotides (length ≥20) in between repeat motifs. Spacers then were grouped according to their trinucleotide content as previously described [11]. For each subject and sample type evaluated, a database of spacer groups was generated, and databases were compared to determine shared spacer groups to create heat maps using Java Treeview [49]. Beta diversity was determined using binary Sorensen distances and was used as input for principal coordinates analysis using Qiime [50]. Spacers from each subject were subjected to BLASTN analysis based on NCBI NR database. Hits were considered significant based on bit scores ≥45, which roughly correlates to 2 nucleotide differences over the 30 nucleotide average length of the spacers, and results displayed using Cytoscape [51]. CRISPR spacers were mapped to each of the bacteriophage, plasmids, and genomes, using CLC Genomics Workbench 4.65 (CLC bio, Boston, MA) using the default parameters for short-read mapping. Circular genome maps were created using CGView [52] and the mapped reads from each set of CRISPR spacers superimposed to scale on the prophage portions of each genome. CRISPR spacer matches to virome reads were defined as exact matches to any spacer within any spacer group. Matches also could be present on either the sequenced strand for each virome read, or its reverse complement. CRISPR spacers for each subject and biogeographic site were used to search all of the virome reads for matches, and the number of spacer matches per read was used to create heatmaps using Java Treeview [49].

### Statistical analysis

To assess whether virome reads or spacer groups had significant overlap between different individuals or biogeographic sites, we performed a permutation test. We simulated the distribution of the fraction of overlapping reads between different individuals or biogeographic sites. For each set, we computed the summed fraction of randomly chosen spacer groups or virome reads, and from those computed an empirical null distribution of statistics. The fraction computed resulted from 10,000 iterations for both spacer groups and virome reads. For the CRISPR spacer groups, 1000 spacer groups were sampled in each iteration, and 10,000 reads were sampled in each iteration for the virome reads. The standard deviation was computed from the percentage of homologous virome reads or spacer groups over the 10,000 iterations. For each subject or biogeographic site, an empirical null distribution of statistics was determined. The observed statistic was referred to this distribution, and the *p* value was computed as the fraction of times the simulated statistic for intra-subject or intra-site comparisons exceeded the simulated statistic for the inter-subject or inter-site comparisons.

### Availability of supporting data

Virome and 16S rRNA sequences are available for download in the MG-RAST database (http://metagenomics.anl.gov/) under project #3928, entitled ‘Dental Plaque Study’.

## Abbreviations

CRISPRs: Clustered regularly interspaced short palindromic repeats; VLP: Virus-like particle; NR: Non-redundant.

## Competing interests

The authors declare that they have no competing interests.

## Authors’ contributions

Conceived and designed experiments: DTP. Performed the experiments DTP, MN, RRS and SRA. Analyzed the data: DTP and SRA. Collected specimens: TKB. Wrote the manuscript: DTP. All authors have read and approved the final manuscript.

## Supplementary Material

Additional file 1: Table S1 Subject demographics. **Table S2.** International tooth enumeration. **Table S3.** Virome reads from each subject. **Table S4.** 16S rRNA reads from each subject. **Table S5.** CRISPR repeat motifs in different streptococci. **Table S6.** Streptococcal Group I (SGI) CRISPRs from each subject. **Table S7.** Streptococcal Group II (SGII) CRISPRs from each subject. **Table S8.** CRISPR spacer homologues. **Table S9.** CRISPR Repeat Motifs and Primers. **Table S10.** Barcode adaptors for primers. Click here for file

Additional file 2: Figure S1 Epifluorescence microscopy of virus-like particles (VLPs) from saliva and dental plaque. **Figure S2.** Percentage of virome contigs with homologues in the NR database. **Figure S3.** Mapping of virome reads to the CRISPR locus of *Streptococcus gordonii* Challis CH1. **Figure S4.** Virome read mappings from all subjects to the CRISPR loci of various *Streptococcus thermophilus* isolates. **Figure S5.** Rarefaction analysis of 16S rRNA from all samples. **Figure S6.** Plots of the trinucleotide difference for SGI (Panel A) and SGII (Panel B) CRISPR spacers. **Figure S7.** Read mapping of CRISPR spacers to *Streptococcus mitis* B6 (Panels A and B), and *S. pneumoniae* 670-6B (Panels C and D). Click here for file
