# Supplementary material for: Characterization of bacteriophage communities and CRISPR profiles from dental plaque
Source: BMC Microbiol. 2014 Jun 30;14:175. doi: 10.1186/1471-2180-14-175 (PMC4104742; doi:10.1186/1471-2180-14-175)
Supplement: Additional file 1: Table S1 — Subject demographics. Table S2. International tooth enumeration. Table S3. Virome reads from each subject. Table S4. 16S rRNA reads from each subject. Table S5. CRISPR repeat motifs in different streptococci. Table S6. Streptococcal Group I (SGI) CRISPRs from each subject. Table S7. Streptococcal Group II (SGII) CRISPRs from each subject. Table S8. CRISPR spacer homologues. Table S9. CRISPR Repeat Motifs and Primers. Table S10. Barcode adaptors for primers. [file 1471-2180-14-175-S1.pdf]

Table S1
Subject demographics

	Age	Gender	Race/Ethnicity
**Subject 1**	35	Male	African American
**Subject 2**	31	Female	Asian
**Subject 3**	24	Female	Asian
**Subject 4**	29	Male	Caucasian

Table S2. International tooth enumeration

Tooth Number	International Enumeration
1	1.8
2	1.7
3	1.6
4	1.5
5	1.4
6	1.3
7	1.2
8	1.1
9	2.1
10	2.2
11	2.3
12	2.4
13	2.5
14	2.6
15	2.7
16	2.8
17	3.8
18	3.7
19	3.6
20	3.5
21	3.4
22	3.3
23	3.2
24	3.1
25	4.1
26	4.2
27	4.3
28	4.4
29	4.5
30	4.6
31	4.7
32	4.8

Table S3
Virome reads from each subject

	Reads							Contigs	
	Initial Count	Trimmed[b]	Low Complexity[c]	Final Count	Length[a]	16S rRNA	Human	Number	Length[a]
Subject 1									
Saliva	1,829,696	587,398	918	1,241,380	96	0	0	181	616
Plaque	721,943	99,308	0	622,635	73	0	0	676	735
Subject 2									
Saliva	1,257,920	620,965	444	636,511	85	0	0	2,094	606
Plaque	2,660,190	801,870	893	1,857,427	91	0	2	970	912
Subject 3									
Saliva	941,661	389,494	1,502	544,665	89	0	0	735	646
Plaque	2,668,766	1,503,124	1,140	1,164,502	93	0	721	1,543	695
Subject 4									
Saliva	1,295,882	536,633	102	759,147	96	0	0	838	716
Plaque	995,619	53,635	0	941,984	87	0	0	673	716

[a]Mean length

[b]Number of reads removed based on quality scores

[c]Reads removed based on multiple homopolymers

Table S4
16S rRNA reads from each subject

	Initial Count	Removed[a]	Final Count	Mean Length
Subject 1				
Saliva	23,628	9,062	14,566	172
Plaque	40,766	24,022	16,744	167
Subject 2				
Saliva	29,757	17,672	12,085	169
Plaque	38,000	19,745	18,255	165
Subject 3				
Saliva	51,090	32,895	18,195	173
Plaque	102,756	57,051	45,705	169
Subject 4				
Saliva	53,380	19,325	34,055	175
Plaque	82,079	50,964	24,779	164

[a]Removed based on length, ambiguity, quality, and homopolymers

Table S5. CRISPR repeat motifs in different streptococci

SGII	SGI
S. gordonii	*S. mutans*
S. thermophilus	*S. thermophilus*
S. gallolyticus	*S. gallolyticus*
S. anginosus	*S. anginosus*
S. salivarius	*S. salivarius*
S. intermedius	*S. infantarius*
S. suis	*S. mitis*
S. pasteurianus	*S. sanguinis*
S. macedonicus	*S. pyogenes*
	S. agalactiae
	S. dysgalactiae
	S. equi

Table S6
Streptococcal Group I (SGI) CRISPRs from each subject

		Number of Reads	Number of Spacers	Spacer Groups	Mean Length	Median Length
Subject 1						
	Saliva	43,466	25,465	207	30	30
	Plaque	93,762	53,974	356	30	30
	All Combined[a]	137,228	79,439	306	30	30
Subject 2						
	Saliva	48,646	25,535	256	30	30
	Plaque	111,734	65,717	289	30	30
	All Combined[a]	160,380	91,252	349	30	30
Subject 3						
	Saliva	31,479	16,844	388	30	30
	Plaque	92,001	45,757	467	30	30
	All Combined[a]	123,480	62,601	473	30	30
Subject 4						
	Saliva	143,840	25,492	193	30	30
	Plaque	121,286	34,355	301	30	30
	All Combined[a]	265,126	59,847	428	30	30
All Subjects						
	Saliva	267,431	93,336	957	30	30
	Plaque	418,783	199,803	1,251	30	30
	All Combined[a]	686,214	293,139	1,912	30	30

[a]Determined by combining all spacers prior to binning based on trinucleotide content

Table S7

Streptococcal Group II (SGII) CRISPRs from each subject

		Number of Reads	Number of Spacers	Spacer Groups	Mean Length	Median Length
Subject 1						
	Saliva	81,466	24,178	105	29	30
	Plaque	121,592	36,065	378	29	30
	All Combined[a]	203,058	60,243	284	29	29
Subject 2						
	Saliva	25,672	16,804	316	30	30
	Plaque	153,425	32,117	469	29	29
	All Combined[a]	179,097	48,921	384	29	29
Subject 3						
	Saliva	27,636	16,172	254	29	30
	Plaque	128,471	53,427	509	30	30
	All Combined[a]	156,107	69,599	439	30	30
Subject 4						
	Saliva	40,068	15,694	199	29	30
	Plaque	220,116	34,652	180	30	30
	All Combined[a]	260,184	50,346	270	30	30
All Subjects						
	Saliva	174,842	72,842	750	29	30
	Plaque	623,604	156,261	1,272	29	30
	All Combined[a]	798,446	229,103	1,084	29	30

[a]Determined by combining all spacers prior to binning based on trinucleotide content

Table S8 - CRISPR spacer homologues

	Streptococcal Group II								Streptococcal Group I							
Sample Type	Saliva	Plaque	Saliva	Plaque	Saliva	Plaque	Saliva	Plaque	Saliva	Plaque	Saliva	Plaque	Saliva	Plaque	Saliva	Plaque
Subject Number	1	1	2	2	3	3	4	4	1	1	2	2	3	3	4	4
**Streptococcal Phage**																
S. phage 040922	0	43	12	2	1	24	26	17	0	4	1	0	0	0	7	0
S. phage 11865	0	0	0	1	1	0	2	1	0	1	0	0	2	1	0	0
S. phage 2167	0	2	1	8	5	24	5	5	11	6	3	1	3	10	0	0
S. phage 34117	0	0	0	5	2	3	2	0	4	1	0	0	6	0	0	0
S. phage 5093	0	0	0	0	0	0	0	0	0	6	0	0	0	0	0	2
S. phage 7201	0	0	0	0	0	0	0	0	0	14	0	1	0	0	0	0
S. phage 8140	0	0	0	30	9	6	4	2	0	0	1	18	0	0	0	0
S. phage 858	0	2	0	0	0	0	0	0	0	0	0	1	0	0	0	0
S. phage Abc2	0	0	0	0	4	0	0	1	1	0	0	0	0	0	0	0
S. phage ALQ13.2	0	0	7	0	0	0	0	0	0	2	0	2	0	0	0	3
S. phage CP1	19	3	4	11	0	57	4	14	2	31	5	6	10	41	38	29
S. phage CP7	0	0	0	0	0	7	0	0	0	0	0	0	0	0	0	0
S. phage DP1	6	26	10	15	1	5	12	2	0	22	12	9	7	4	0	0
S. phage DT1	0	0	0	0	0	0	0	0	0	1	0	0	0	0	0	0
S. phage DT1.1	0	0	0	0	0	0	0	0	0	4	0	0	0	0	0	0
S. phage EJ1	0	1	0	11	1	11	1	50	0	3	0	2	0	4	11	11
S. phage M102	0	1	0	0	0	0	0	0	0	0	0	0	0	0	0	0
S. phage MD2	0	1	0	0	0	0	0	0	0	0	0	0	0	0	0	0
S. phage MM1 1998	0	0	0	0	0	1	0	1	0	0	0	0	0	0	0	0
S. phage PH10	2	42	15	56	67	38	24	26	0	13	20	60	13	37	10	0
S. phage PH15	0	40	9	1	0	8	14	16	0	2	0	0	1	0	1	0
S. phage Sfi11	0	0	0	1	0	0	0	1	1	0	0	2	0	0	0	0
S. phage SM1	4	0	2	61	2	30	19	13	3	21	45	40	4	51	8	26
S. phage SMP	0	0	0	0	0	3	0	0	0	0	0	0	15	4	0	0
S. phage V22	0	0	0	2	2	20	3	0	0	5	0	0	0	1	5	25
S. phage YMC-2011	0	4	1	9	0	0	0	0	0	3	0	2	2	0	2	6

Streptococcal Plasmids and Transposons

S. plasmid 1	0	0	0	0	1	4	4	6	0	0	0	1	0	0	0	0
S. plasmid pDRPIS7493	0	0	0	0	0	0	0	0	0	0	0	0	0	2	7	22
S. plasmid pFW213	2	0	0	0	0	0	0	0	0	2	1	0	0	0	0	0
S. plasmid pND103	0	0	0	1	0	0	0	0	0	0	0	0	0	0	0	0
S. plasmid pSMQ173b	0	0	0	0	0	1	1	4	0	0	0	0	0	0	0	0
S. plasmid pSMQ-316	0	0	0	0	0	0	0	0	0	0	0	0	0	0	0	1
S. plasmid pSpnP1	74	3	1	1	0	0	0	42	0	21	0	0	0	43	6	24
S. transposon Tn1311	0	0	0	0	0	0	0	0	0	0	0	0	0	0	2	0

Streptococcal Genomes

S. gallolyticus ATCC BAA-2069	0	0	2	0	0	0	0	0	0	0	0	0	0	0	0	0
S. gallolyticus UCN34	0	0	0	4	0	0	0	0	0	0	0	5	0	0	0	0
S. gordonii CH1	0	0	0	0	0	5	8	15	0	0	0	0	0	0	0	0
S. mitis B6 complete genome	0	0	0	55	4	17	2	81	0	0	4	6	3	1	1	1
S. mutans UA159	0	0	1	0	0	0	0	0	0	0	0	0	0	0	0	0
S. oralis Uo5	0	0	0	0	0	2	0	3	0	0	0	0	0	0	0	0
S. parasanguinis ATCC 15912	0	0	0	0	0	0	0	2	0	0	0	0	0	0	0	13
S. pneumoniae 19A-6	0	1	0	1	1	0	1	1	0	0	0	1	0	0	0	0
S. pneumoniae 670-6B	0	1	0	1	0	12	8	16	0	0	0	0	1	0	0	0
S. pneumoniae 70585	0	0	0	0	0	1	0	0	0	0	0	0	2	1	1	0
S. pneumoniae AP200	0	0	0	1	0	16	14	8	0	5	1	1	1	3	6	8
S. pneumoniae ATCC 700669	7	0	0	5	0	3	0	0	0	4	3	3	1	2	1	0
S. pneumoniae G54	0	0	0	6	0	0	1	1	0	0	0	2	0	0	0	0
S. pneumoniae JJA	0	0	0	4	1	0	0	0	0	0	1	2	0	0	1	0
S. pneumoniae OXC141	0	0	0	1	0	17	3	3	12	3	2	0	10	2	0	0
S. pneumoniae P1031	0	0	0	2	0	0	1	1	0	0	1	0	0	0	0	0
S. pseudopneumoniae IS7493	0	12	1	84	4	25	4	18	20	21	4	5	11	7	11	13
S. salivarius JIM8777	0	14	1	24	1	0	0	5	0	0	0	16	2	0	30	0
S. thermophilus DGCC1443	0	0	0	2	0	0	0	0	0	3	0	1	0	0	1	0
S. thermophilus JIM 8229	0	0	0	0	0	0	0	0	0	10	0	0	0	0	0	0
S. thermophilus DGCC 7710	0	0	0	0	0	1	0	1	0	0	0	0	0	0	0	0
S. thermophilus DGCC292	0	0	0	0	0	0	0	0	0	0	0	0	0	0	0	1
S. thermophilus DGCC66	0	0	0	0	0	0	0	0	0	0	1	0	0	0	0	0

S. thermophilus DGCC766	0	0	0	5	0	1	0	0	0	5	0	3	0	0	0	10
S. thermophilus DGCC7796	0	0	0	0	0	0	0	0	0	0	0	0	2	1	0	145
S. thermophilus DGCC7809	0	0	0	0	0	0	0	0	0	0	0	1	0	0	0	11
S. thermophilus DGCC7984	0	0	0	1	0	0	0	0	0	0	0	0	0	0	0	0
S. thermophilus JIM 8230	0	0	0	0	0	0	0	0	0	0	0	0	1	0	0	0
S. thermophilus LMG 18311	0	0	0	0	0	2	2	1	0	0	0	0	0	0	0	0
S. thermophilus ND03	0	0	0	0	0	0	0	0	1	4	0	0	0	0	0	0
S. thermophilus WTphi858phi297	0	0	0	0	0	2	0	0	0	13	0	1	0	2	0	8

Non Streptococci

Bacillus cereus 03BB102	0	0	0	0	0	0	0	1	0	0	0	0	0	0	0	0
Bacillus cereus AH820	0	0	0	0	0	1	0	0	0	0	0	0	0	0	0	0
Bacillus plasmid WSH-002	0	2	0	0	0	0	0	0	0	0	0	0	0	0	0	0
Cellulophaga algicola DSM 14237	0	1	0	0	0	0	0	0	0	0	0	0	0	0	0	0
Clostridium botulinum E3	0	0	0	3	0	0	0	0	0	4	3	6	0	4	1	0
Clostridium difficile BI1	0	0	0	0	0	0	0	1	0	0	0	0	0	0	0	0
Clostridium difficile M120	0	1	1	0	0	0	0	0	0	0	0	0	0	0	0	0
Clostridium tetani E88	0	0	0	0	0	0	0	0	0	1	1	0	0	0	0	0
Cyanothece sp. PCC 7822	0	0	0	0	0	3	0	0	0	0	0	0	0	1	0	0
Desulfobacterium sp.	0	0	0	0	0	0	1	0	0	0	0	0	0	0	0	0
Eubacterium limosum KIST612	0	0	0	0	0	0	0	0	0	0	1	1	0	0	0	0
Fusobacterium nucleatum ATCC 255	0	0	0	1	0	0	0	0	0	0	0	0	0	0	0	0
Geobacillus sp. WCH70	0	0	0	0	0	0	0	0	0	1	0	0	0	0	0	0
Lactobacillus delbrueckii ND02	0	0	0	0	0	0	0	0	0	0	0	1	0	0	0	0
Lactobacillus salivarius CECT 5713	0	0	0	0	0	0	0	1	0	0	0	0	0	0	0	0
Listeria monocytogenes Clip80459	0	0	0	0	0	0	0	0	1	0	0	0	0	0	0	0
Listeria monocytogenes FSL R2-561	0	0	0	0	0	0	1	0	0	0	0	0	0	0	0	0
Little cherry virus 2 USA6b	0	0	0	0	0	0	0	0	0	0	1	0	0	0	0	0
Lysinibacillus sphaericus C3-41	0	0	0	0	0	0	0	0	0	0	0	1	0	0	0	0
Marinobacter adhaerens HP15	0	0	0	0	0	0	0	0	0	0	1	0	0	0	0	0
Neisseria gonorrheae MS11	0	2	1	0	0	6	0	0	0	0	0	0	2	6	0	0
Pediococcus pentosaceus ATCC 25745	0	0	0	0	0	2	1	2	0	0	0	0	0	0	0	0

Rhodopirellula baltica SH1	0	0	0	0	0	0	0	0	0	0	0	1	0	0	0	0
Shewanella sp. W3-18-1	0	1	12	1	0	0	0	0	6	12	0	6	0	0	0	0
Uncultured marine bacterium	0	0	0	0	0	0	0	0	0	0	0	0	1	0	0	0
Uncultured marine bacterium MedDCM	0	0	0	0	0	2	0	0	0	0	0	0	0	0	0	0

Table S9. CRISPR Repeat Motifs and Primers

**Name**		
**Streptococcus (SGII)**[a]	Consensus	GTTGTACAGTTACTTAAATCTTGAGAGTACAAAAAC
	Forward	CCATCTCATCCCTGCGTGTCTCCGACTCAGXXXXXXXXXXCAGTTACTTAAATCTTGAGAG
	Reverse	CCTCTCTATGGGCAGTCGGTGATAGATTTAAGTAACTGTACAAC
**Streptococcus (SGI)**[b]	Consensus	GTTTTGGAACCATTCGAAACAACACAGCTCTAAAAC
	Forward	CCATCTCATCCCTGCGTGTCTCCGACTCAGXXXXXXXXXXCGTTTACGGTATTGAAAC
	Reverse	CCTCTCTATGGGCAGTCGGTGATGGTAAGCACTGTTGCAAC

[a]Identified from Streptococcus gordonii

[b]Identified from Streptococcus mutans

[c]X’s represent the location of the barcode sequence

Table S10

Barcode adaptors for primers

	Barcode Number	Barcode Sequence
Viromes		
	1	ACGAGTGCGT
	2	ACGCTCGACA
	3	AGACGCACTC
	4	AGCACTGTAG
	5	ATCAGACACG
	6	ATATCGCGAG
	7	CGTGTCTCTA
	8	CTCGCGTGTC
	9	TAGTATCAGC
	10	TCTCTATGCG
	11	TGATACGTCT
16S rRNA or CRISPRs		
	1	ACGAGTGCGT
	2	ACGCTCGACA
	3	AGACGCACTC
	4	AGCACTGTAG
	5	ATCAGACACG
	6	ATATCGCGAG
	7	CGTGTCTCTA
	8	CTCGCGTGTC
	9	TAGTATCAGC
	10	TCTCTATGCG
	11	TGATACGTCT
	12	TACTGAGCTA
	13	CATAGTAGTG
	14	CGAGAGATAC
	15	ATACGACGTA
	16	TCACGTACTA
	17	CGTCTAGTAC
	18	TCTACGTAGC
	19	TGTACTACTC
	20	ACGACTACAG
	21	CGTAGACTAG
	22	TACGAGTATG
	23	TACTCTCGTG
