# Supplementary material for: Characterization of bacteriophage communities and CRISPR profiles from dental plaque
Source: BMC Microbiol. 2014 Jun 30;14:175. doi: 10.1186/1471-2180-14-175 (PMC4104742; doi:10.1186/1471-2180-14-175)
Supplement: Additional file 2: Figure S1 — Epifluorescence microscopy of virus-like particles (VLPs) from saliva and dental plaque. Figure S2. Percentage of virome contigs with homologues in the NR database. Figure S3. Mapping of virome reads to the CRISPR locus of Streptococcus gordonii Challis CH1. Figure S4. Virome read mappings from all subjects to the CRISPR loci of various Streptococcus thermophilus isolates. Figure S5. Rarefaction analysis of 16S rRNA from all samples. Figure S6. Plots of the trinucleotide difference for SGI (Panel A) and SGII (Panel B) CRISPR spacers. Figure S7. Read mapping of CRISPR spacers to Streptococcus mitis B6 (Panels A and B), and S. pneumoniae 670-6B (Panels C and D). [file 1471-2180-14-175-S2.pdf]

# Figure S1

A.

B.

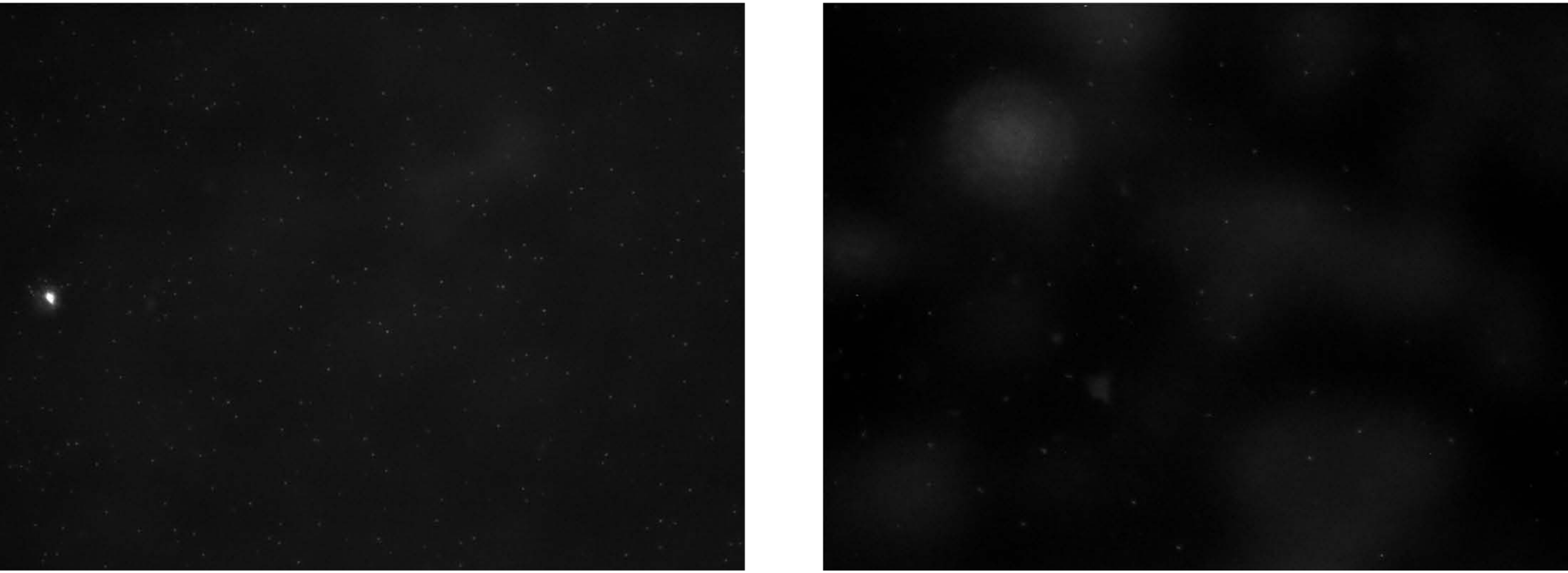

**Figure S1**: Epifluorescence microscopy of virus-like particles from the saliva (Panel A) and pooled subgingival plaque (Panel B) of subject #1. Virus-like particles were present at $10^8$ per milliliter of saliva and $10^{10}$ per gram of pooled dental plaque.

# Figure S2

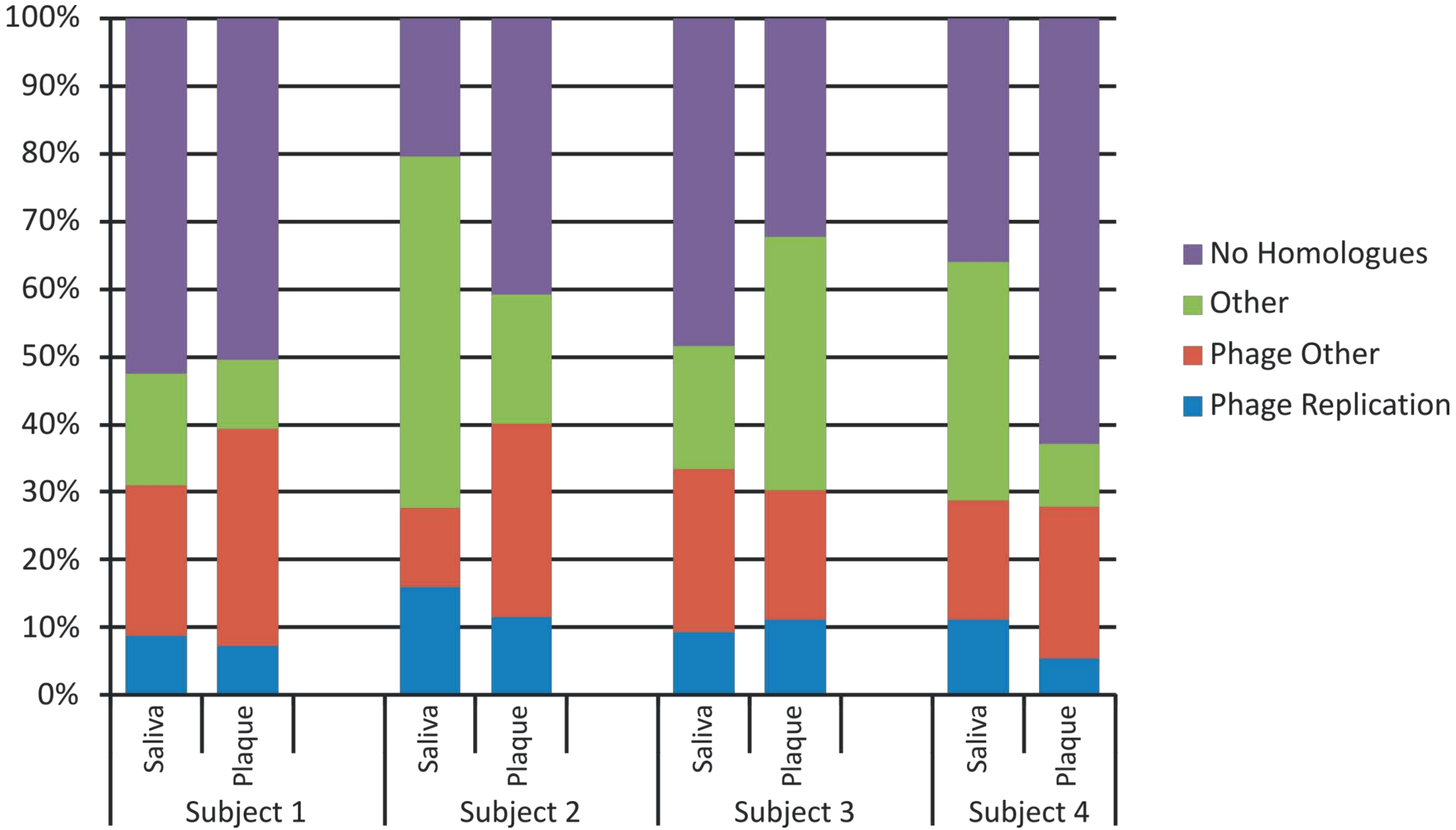


**Figure S2**: Percentage of virome contigs assigned to various categories. Those contigs marked as 'Phage Other' generally denote structural and virulence genes in known phage, and those marked 'Other' generally represent contigs homologous to bacterial genomes.

# Figure S3

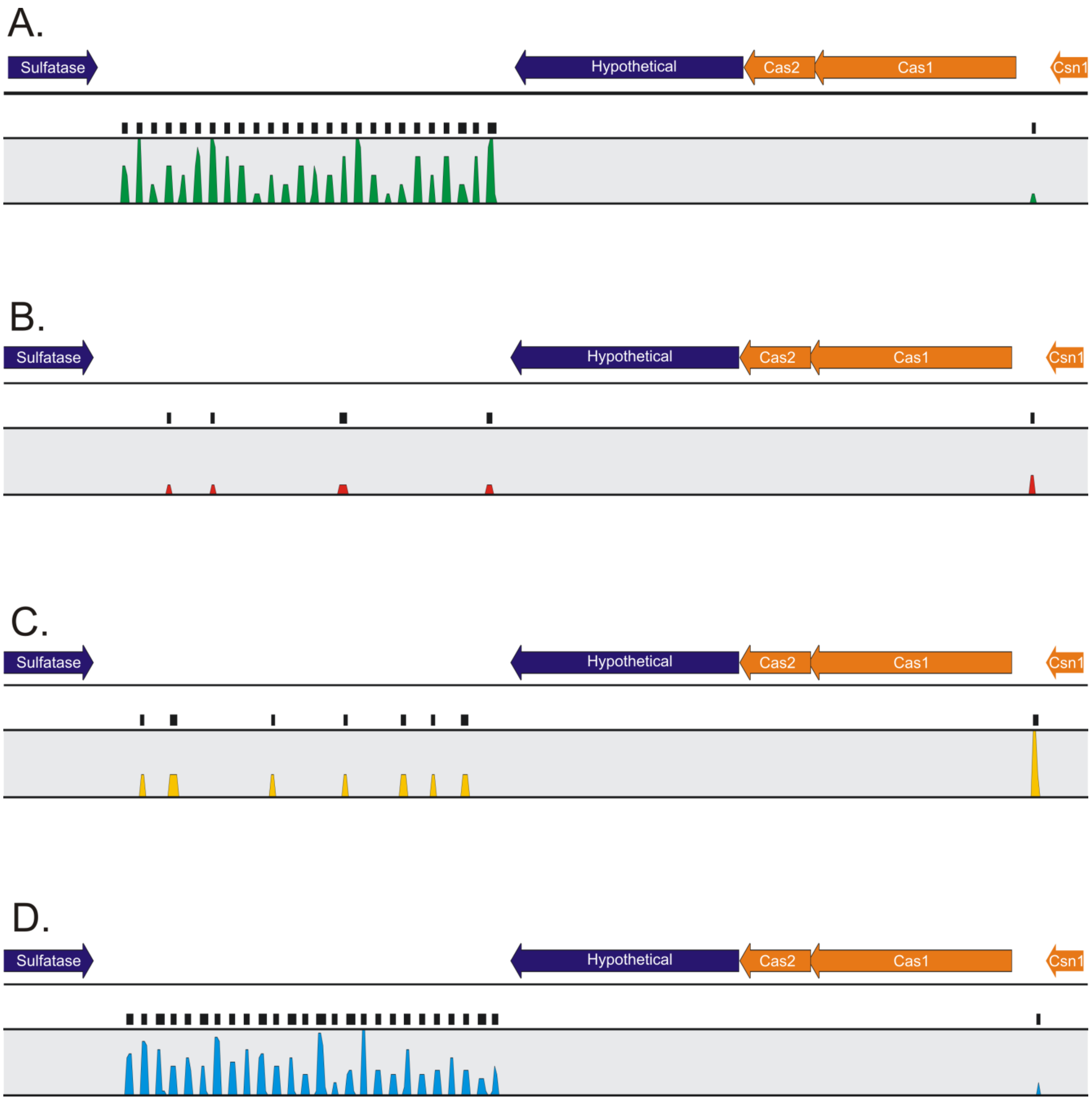


**Figure S3**: Mapping of virome reads from each subject to the CRISPR locus of *Streptococcus gordonii* Challis CH1. Genes flanking the CRISPR locus are demonstrated by arrows, and portions of the CRISPR locus where reads map are demonstrated by the colored peaks. The Y-axis is scaled from 1 to 16. For Subjects #1 and #4, all *S. gordonii* CRISPR spacers in the CRISPR locus were matched by viruses present in these viromes. No CRISPR repeat sequences were represented in any of the viromes. Panel A - subject #1, Panel B - subject #2, Panel C - subject #3, and Panel D - subject #4.

**Figure S4**

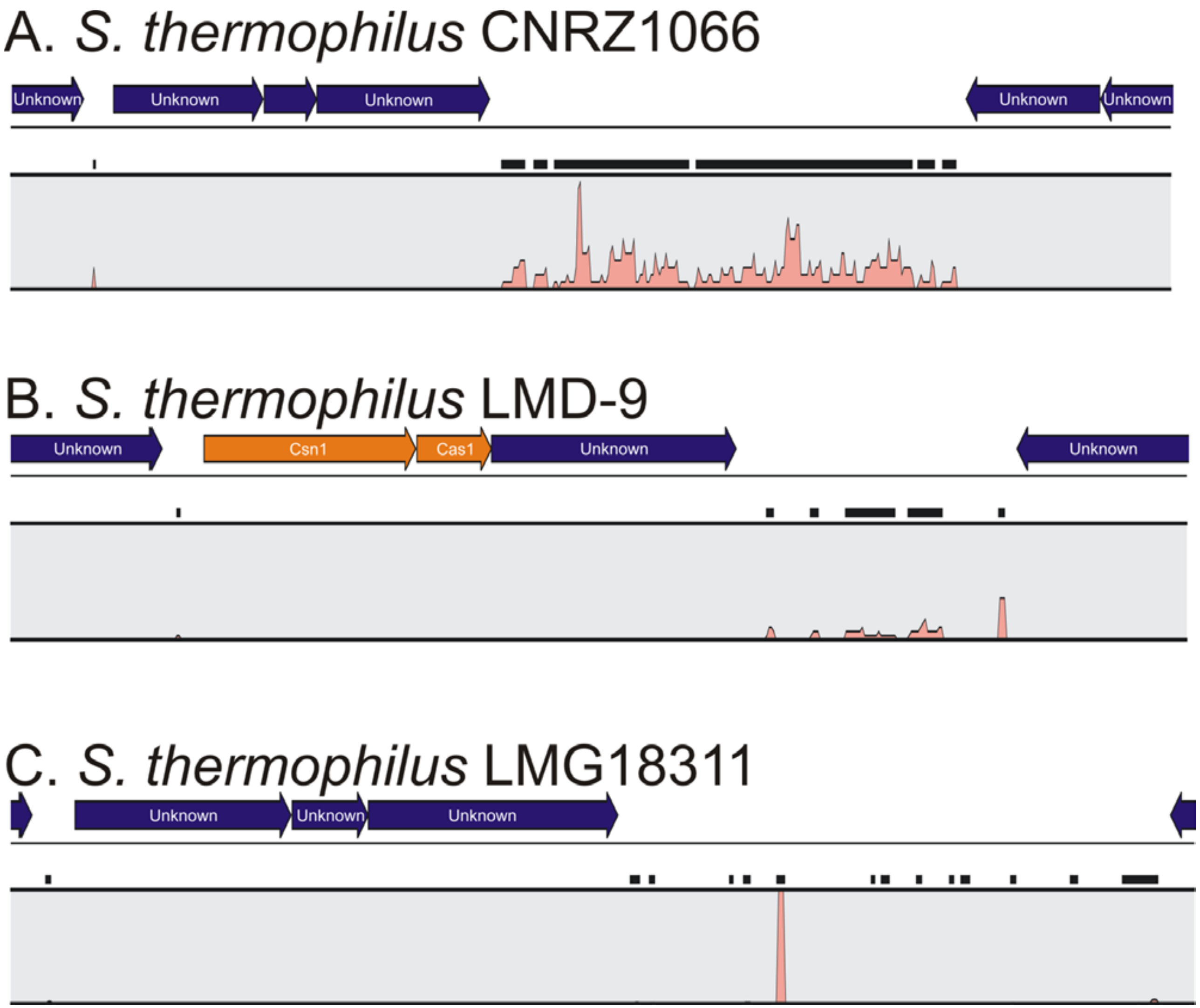


**Figure S4**: Mapping of virome reads from all subjects combined to the CRISPR loci of *Streptococcus thermophilus* isolates. Genes flanking the CRISPR locus are demonstrated by arrows, and portions of the CRISPR locus where reads map are demonstrated by the colored peaks. No CRISPR repeat sequences were represented in any of the viromes. Panel A - *S. thermophilus* CNRZ1066 with the Y-axis scaled to 16, Panel B - *S. thermophilus* LMD-9 with the Y-axis scaled to 28, and Panel C - *S. thermophilus* LMG18311 with the Y-axis scaled to 284.

**Figure S5**

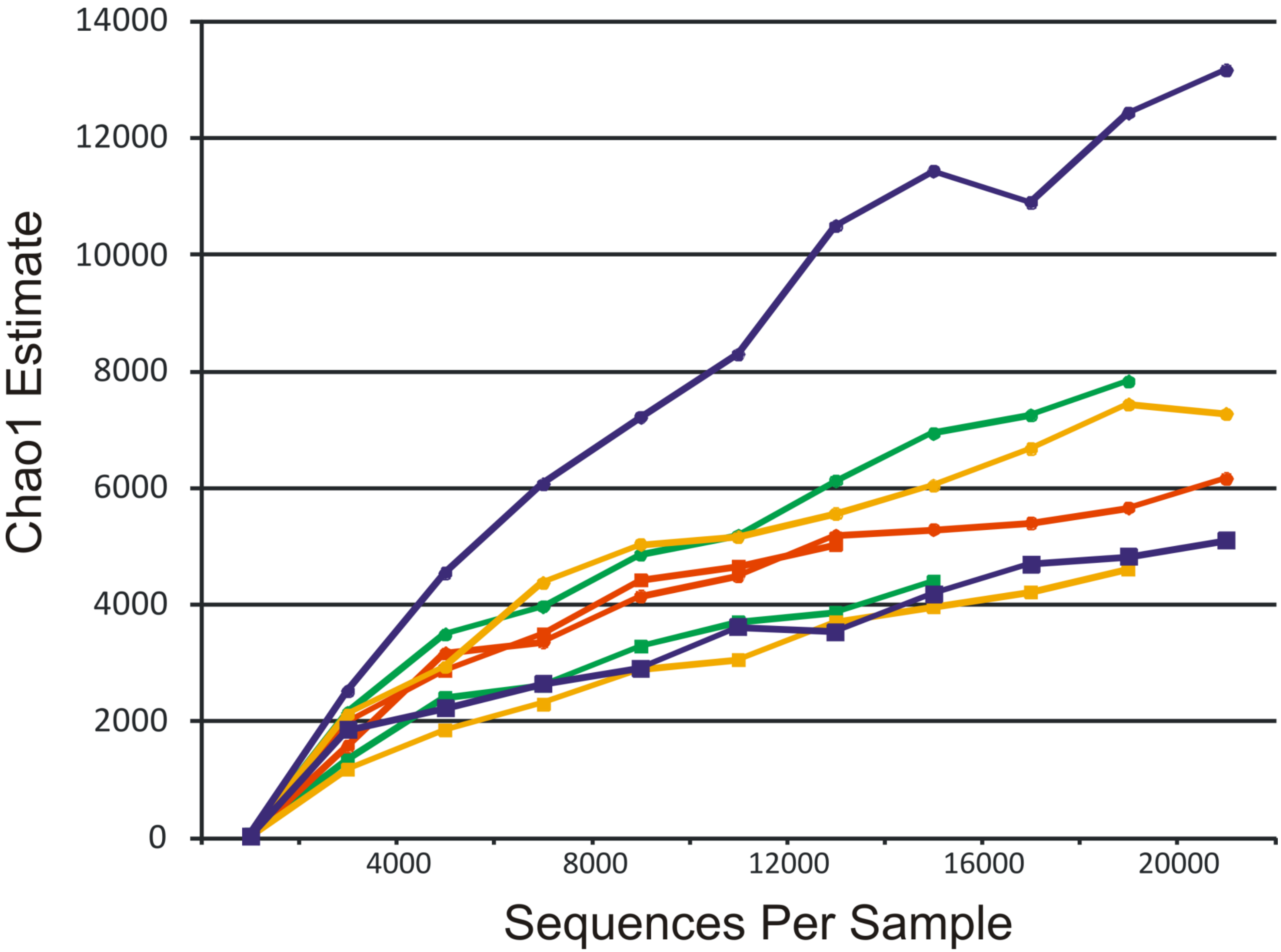


**Figure S5**: Rarefaction analysis of 16S rRNA from all subjects and sample types. Subject #1 is represented in green, subject #2 in red, subject #3 in gold, and subject #4 in blue. Saliva is represented by squares and plaque by circles.

**Figure S6**

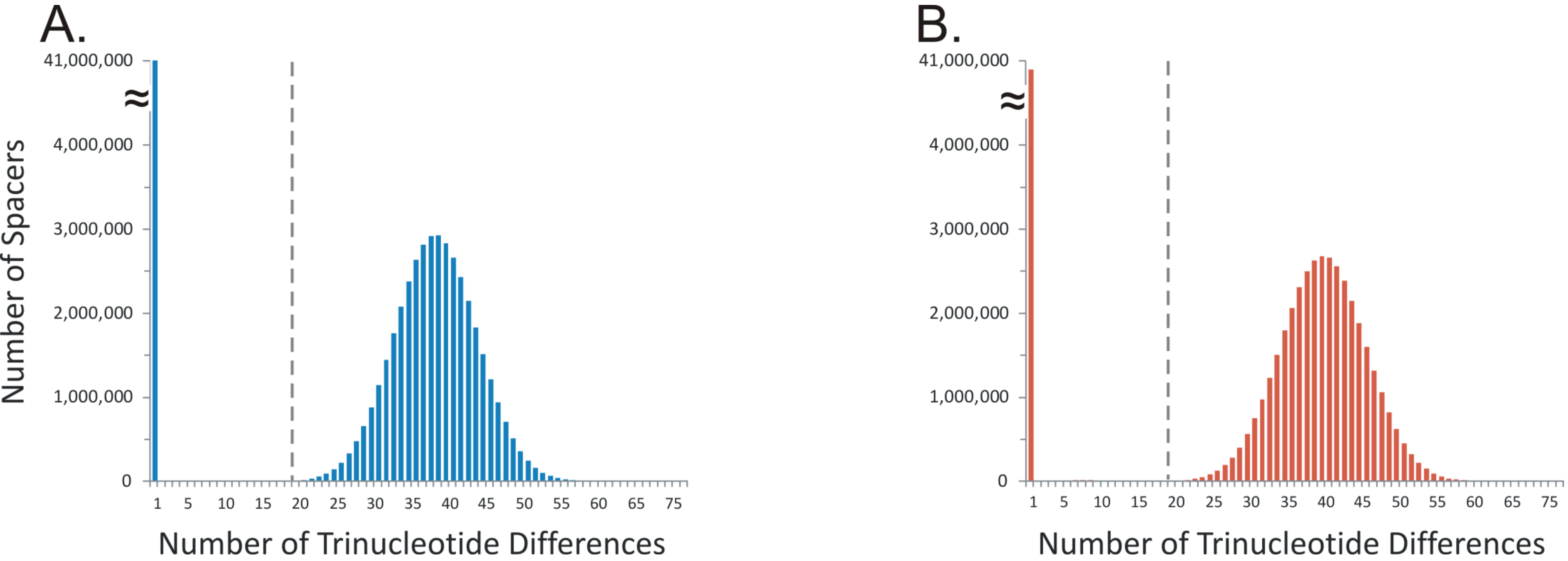


**Figure S6**: Plots of the number of trinucleotide difference for SGI (Panel A) and SGII (Panel B) CRISPR spacers. The dashed line corresponds to the statistical cutoff for binning of spacers into spacer groups.

**Figure S7**

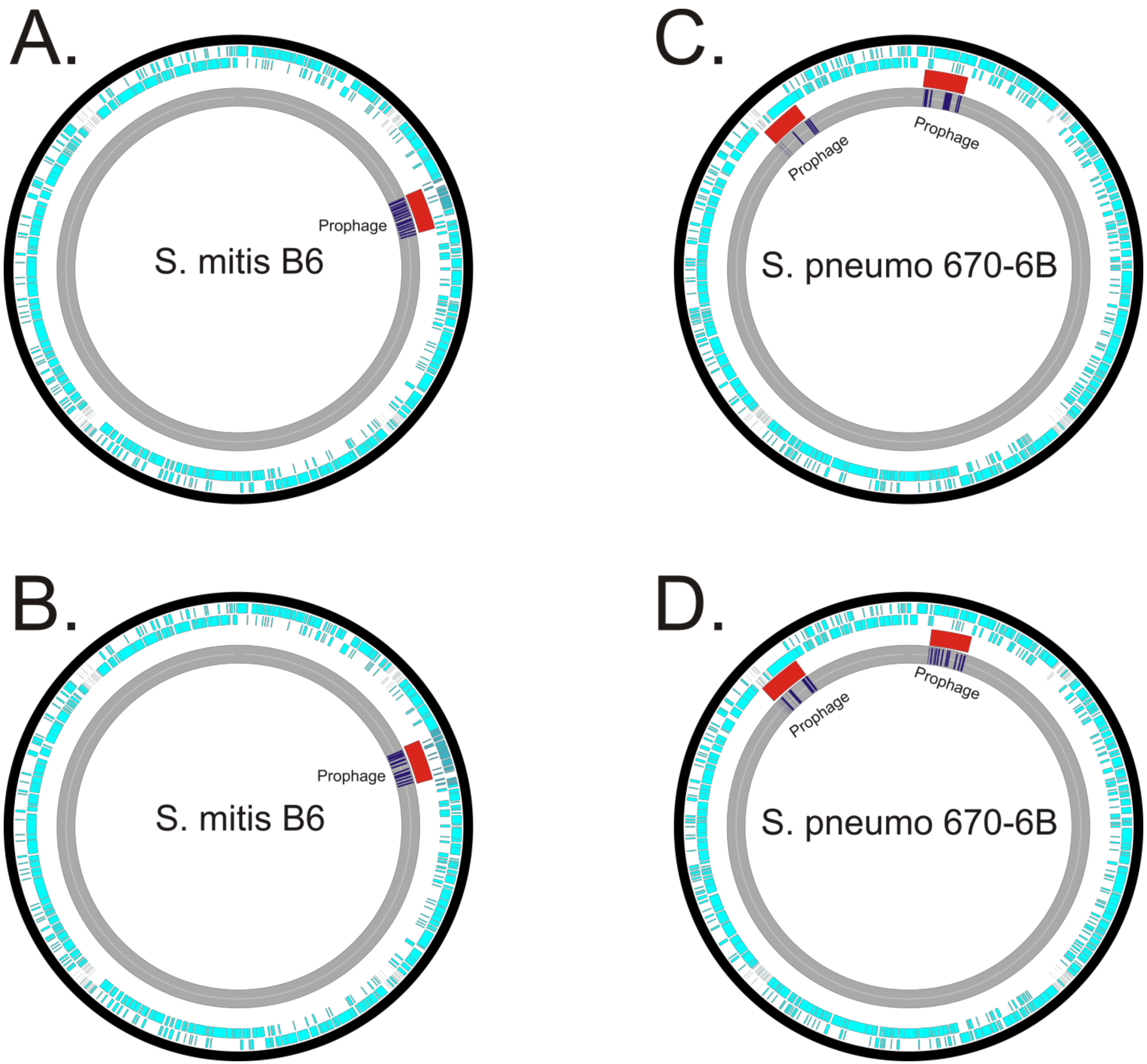


**Figure S7:** Read mapping of CRISPR spacers to *Streptococcus mitis* B6 (Panels A and B), and *S. pneumoniae* 670-6B (Panels C and D). ORFs are shown in cyan, prophage in each genome are represented in red, and CRISPR spacers matching the prophage are represented in blue. Panels A and C represent SGI CRISPR spacers, and Panels B and D represent SGII CRISPR spacers.
